# *Nigella sativa*and its Derivatives as Food Toxicity Protectant Agents

**DOI:** 10.15171/apb.2019.004

**Published:** 2019-02-21

**Authors:** Zahra Karimi, Adel Mirza Alizadeh, Jafar Ezzati Nazhad Dolatabadi, Parvin Dehghan

**Affiliations:** ^1^Immunology Research Center, Tabriz University of Medical Sciences, Tabriz, Iran.; ^2^Department of Food Science and Technology, Nutrition Research Center, Faculty of Nutrition and Food Sciences, Tabriz University of Medical Sciences, Tabriz, Iran.; ^3^Student Research Committee, Tabriz University of Medical Sciences, Tabriz, Iran.; ^4^Student Research Committee, Department of Food Technology, Faculty of Nutrition Sciences and Food Technology/ National Nutrition and Food Technology Research Institute, Shahid Beheshti University of Medical Sciences, Tehran, Iran.; ^5^Drug Applied Research Center, Tabriz University of Medical Sciences, Tabriz, Iran.

**Keywords:** *Nigella sativa*, Thymoquinone, Oxidative stress, Food protectant, Food toxicity

## Abstract

Exposure to food toxins generate multiple adverse health effects. Heavy metals, antibiotics residue, mycotoxins, pesticides and some food additives are examples of the most important food toxins. The common mechanism of toxicity and carcinogenicity effects of food toxins is the generation of oxidative stress that leads to DNA damages. Moreover, based on epidemiologic evidence unhealthy eating habits and food toxicities are associated with cancers occurrence. Therefore, application of bioactive food additives as harmless or safe components in food industry is expensive. *Nigella sativa* L. is a broadly used herb-drug for various diseases all over the world and has been used as preservative and food additive. Based on various studies *N. sativa* has shown various pharmacological activities including therapeutic efficacy against different human diseases and antioxidant anti-inflammatory effects against environmental toxins. *N. sativa* decreases the adverse health effects induced by mentioned food toxins via modulating the action of antioxidant enzymes such as glutathione peroxidase (GPx), glutathione-S-transferase catalase and act as reactive oxygen species (ROS) scavengers in different organs. Besides, *N. sativa* and thymoquinone (TQ) have protective effects on food products through removal and inhibition of various toxic compounds. Therefore, in the present review we will describe all protective effects of *N. sativa* and its main constituents, TQ, against food induced toxicities.

## Introduction

One of the basic human need is achieving safe and nutritious food with the advent of the modern industrial world. Due to current methods in food technology, food products can become contaminated by biological and chemical agents that have the potential to cause side effects on human health. Human diet contains thousands of different chemical agents which only a few of them have nutritional value. Recently, public concerns result in more demand for systematic food safety assessment and efforts to produce healthy and improved food. However, food toxicity may result from chemicals existence that is normal components of plant and animal foodstuffs or enters foods as natural contaminants before of manipulation by human. The US Food and Drug Administration (FDA) has classified the relative significance of health hazards related to food including microbiological contamination, incorrect eating habits, environmental contamination, natural toxic components, pesticide residues, food additives.^1^ Various studies have been done to reduce toxicity and presently, the pharmaceutical and food industries are in exploration of novel composites that are multi-functional, bioactive and harmless.

**Figure 1:**
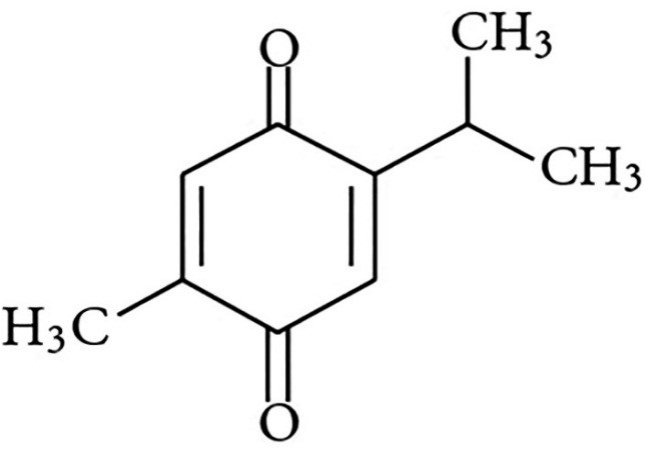
Chemical structure of thymoquinone.

**Figure 2:**
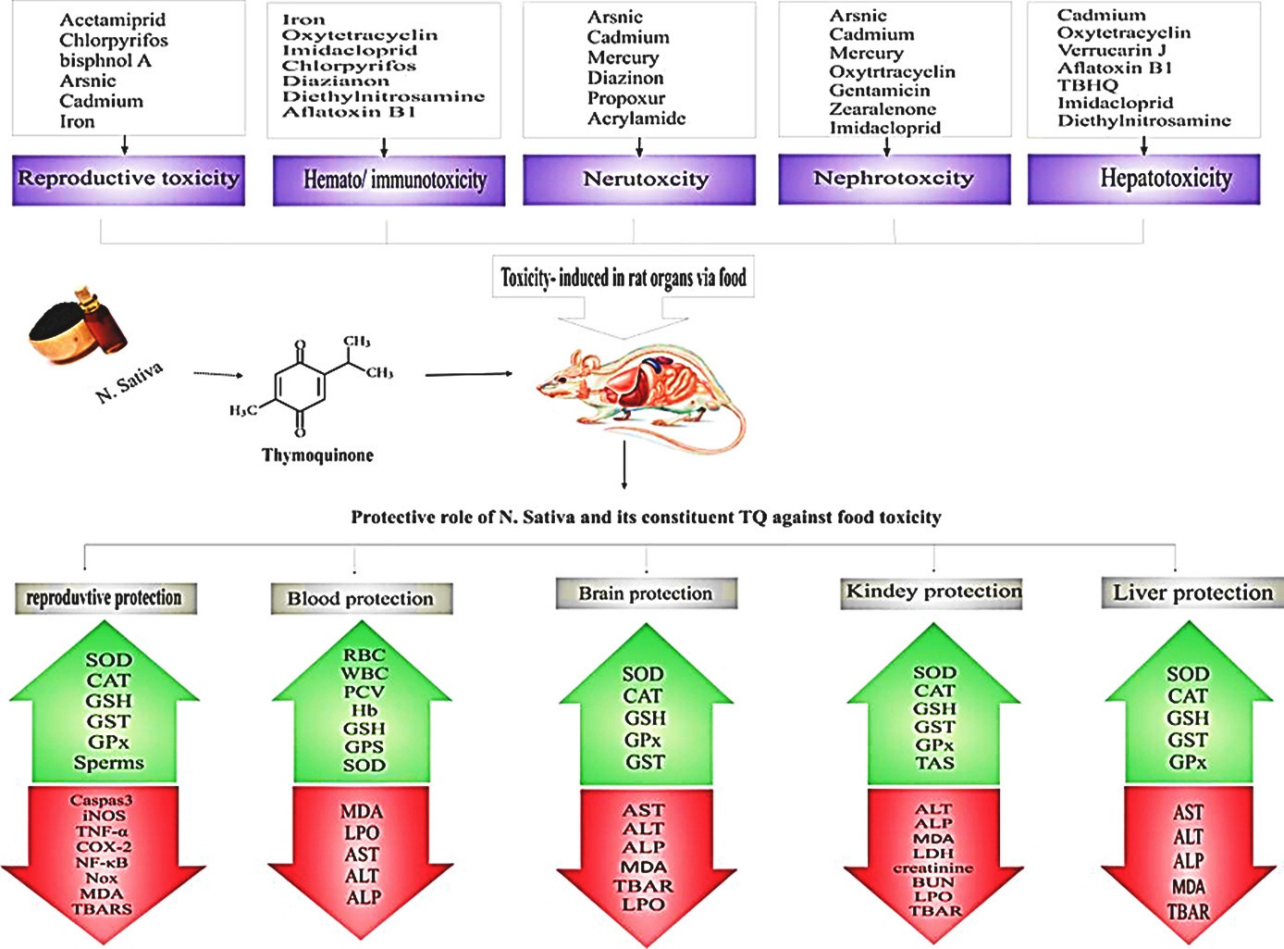
Schematic illustration of *Nigella sativa* its main constituent, TQ, protective effect against food induced toxicities in brain, liver, kidney, blood, genome and reproductive organs.

*Nigella sativa* L. is one of the most admired medicinal herb seeds in traditional medicines. The seeds are the most commonly used parts of the plant in the traditional medicine. Seeds have pungent and bitter taste with considerable amount of oil inside its united follicles, which mostly are utilized as a food preservative and spice and the most often used therapeutic agent is its oil. The seeds are very pleasant with hot, strong, peppery taste and are usually consumed in cooking curries, pastries and Mediterranean cheeses. Also, it can be added to coffee, tea, and bread, canned foods, wine and vinegar.^2^ The black seed oil contains more than 30% fixed oil and 0.4%-0.45% (w/w) volatile oil such as thymoquinone (TQ) (4%–24%) and 46% of monoterpenes such as α-pinene and r-cymene. Ghosheh et al identified 4 main compounds of BS oil using high performance liquid chromatography (HPLC) method including TQ, dithymoquinone, thymohydroquinone, and thymol.^3^ The popularity of *N. sativa* was due to the ideological belief in the herb as a remedy for various diseases. *N. sativa* and its active constituents, TQ, (Figure 1) have been investigated for its biological effects and remedy potential owing to its broad spectrum of activities including antimicrobial, anticancer, immunomodulation, analgesic, anti-inflammatory, spasmolytic, hepatoprotective, renal protective, gastro-protective, antidiabetic, antioxidant and bronchodilator.^4^ In addition, various researchers demonstrated that *N. sativa* and TQ have very low side effects and toxicity. Therefore, in this article, food protective effects of *N. sativa* and its main constituents through inhibition of various toxic compounds which have been generated in food processing steps like heavy metals, antibiotics, mycotoxins, pesticides and food additives both *in vitro* and *in vivo* experimental models are overviewed. Also, the effects of TQ on inhibition of food toxicity induced in different organs including brain, liver, kidney, blood, genome and reproductive tract (Figure 2) have been discussed.

### Protective roles of Nigella sativa and its constituent thymoquinone against heavy metals toxicity

**Figure 3:**
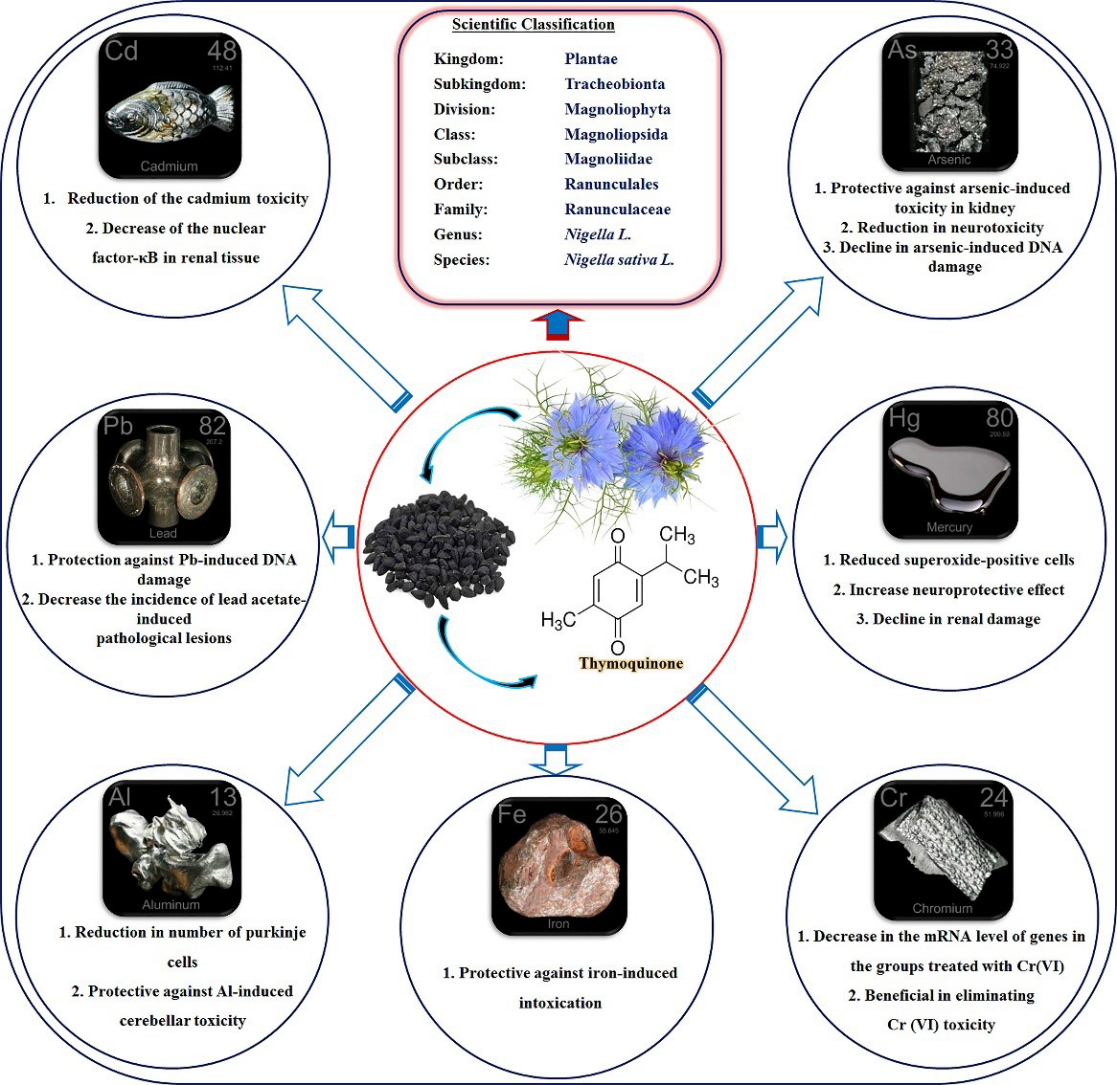
Schematic description of *Nigella sativa* and TQ against toxicities induced by some heavy metals.

Heavy metals such as arsenic (As), aluminum (Al), chromium (Cr), lead (Pb), nickel (Ni), cadmium (Cd), mercury (Hg), etc contribute lots of environmental and health problems based on their toxicity effects. Toxic metals can be exposed to human and environment through numerous ways such as air, food, water, waste, and industries and the accumulation of their ions led to serious environmental and health hazards. There are various methods to remove or reduce the toxicity effect of these heavy metals based on their types. Physical, biological and chemical methods are widely used in the treatment and removal of organic pollutant. Herbal treatment is one of the well-known and reliable methods to decrease chronic and acute toxic effects of organic pollutants like heavy metals in animal and human organs. Meanwhile, medicinal plant like *N. sativa* (black seed) is a widely used as an antidotal and protective agent due to its effective constituent, TQ. There are various reports on its biological activities and protective effects in different organs and tissues including brain, genome, liver, kidney, lung, etc. In this section of the review article, several studies in scientific databases that evaluate the protective effects of *N. sativa* and its main components against natural and chemical-induced toxicities are demonstrated. At the end of this section, the schematic description of *N. sativa* and TQ against toxicities induced by some heavy metals is shown in Figure 3. Besides, Table 1 has shown the effect of *N. sativa* and TQ against heavy metals toxicity.

**Table 1:** Protective effects of *Nigella sativa* and its constituent thymoquinone against some heavy metals in different tissues

**Food Toxins**	**Food Source**	**Targets**	**Treatment Conditions**	**Main Findings**	**Ref.**
Arsenic	Rice, vegetables, seafood	Neurotoxicity	*In vivo*, rat, TQ: 10 µM	The neurotoxicity decrease was due to antioxidant potential of TQ	^5^
		Testicular Toxicity	*In vivo*, rat, TQ: 10 mg/kg/d	TQ could protect against arsenic-induced testicular injury.	^6^
		Renal Failure	*In vivo*, rat, TQ: 10 mg/kg/d	TQ plays a protective role against arsenic-induced toxicity in the kidney.	^7^
		Neurotoxicity	*In vivo*, rat, TQ: 10 mg/kg/d	TQ can reduce the neurotoxic effect of arsenate.	^8^
Cadmium	Shellfish, mushroom , grains	Hepatotoxicity	*In vivo*, mice, TQ: 10 µM	TQ protected against cadmium-induced through its antioxidant oxidative stress.	^9^
Mercury	Fish	Nephrotoxicity	*In vivo*, rat, TQ, 10 mg/kg/d	Antioxidant effect of TQ prevents acute renal failure due to mercury toxicity.	^10^
-	-	Nephrotoxicity	*In vivo*, rat, TQ (5 mg/kg/d, per os);	Protective effect of TQ against Pb-induced renal antioxidant capacity impairment	^11^
		Neurotoxicity	*In vivo*, rat, TQ:20 mg/kg b.w	TQ decreased the incidence of lead acetate-induced pathological lesions.	^12^
Aluminum (AL chloride)	Air, but not in its metallic form, in the food industry, drinking water, pharmaceutica and cooking utensils.	Cerebellar toxicity	*In vivo*, rat, NS oil:(1 ml/kg) orally	*N. sativa* may play a protective role against Al-induced cerebellar toxicity in humans.	^13^
Iron	Water, iron supplement	Hematotoxicity	*In vivo*, Rabbit *N. Sativa* and Oriental Spices	*N. sativa* can be effective for health protection and health enhancement of organism.	^14^
		Reduce toxicity	*In vivo*, rat, TQ: 0.005M	TQ can inhibit the toxicity of some metals.	^15^
Chromium	Leather tanning, electroplating and stainless steel industries	Genotoxicity	*In vivo*, Tilapia and Zebrafish crude extract Nile of *N. sativa*:0.5 mg/kg	Inhibition may be mediated through antioxidant activity and inhibition of Cr(VI) enzymatic activation.	^16^
		Reduce toxicity	*In vivo*. Rat, TQ: 500 µM	TQ can be beneficial for eliminating Cr(VI) toxicity	^17^

### Protective roles against arsenic toxicity

As is an example of the earliest poisons known to human and widely dispersed in its various forms in the atmosphere.^18^ It can contaminated environment via natural weathering of geological forms and human factors as well as mining manufacturing, wash-off, burning of fossil fuels, agricultural and incineration.^19^ An exposure to a high-dose of As may result in severe adverse reactions like diarrhea, pain, vomiting, dehydration and weakness.^18^ Pigmentation changes, liver disease, anemia, and gastrointestinal symptoms, hyperkeratosis, Bowen’s disease, squamous and basal cell carcinoma, diabetes mellitus and Blackfoot disease are chronic symptoms of As exposure.^20,21^ Since As leads in formation constitution of reactive oxygen species (ROS) and induces lipid peroxidation (LPO), ROS-mediated oxidative damage is a common denominator of As pathogenesis that in turn produce active oxygen and nitrile molecule.^22^ TQ can play a considerable mitigatory role against various toxicities that induced with heavy metal such as As.^23^ For example, Firdaus et al have shown that in brain preparations of male Wistar rats, significant reduction in the As-induced neurotoxicity has been observed upon pre-treatment with TQ.^5^ Also, decrease in As-induced DNA damage upon pre-treatment with TQ was approved through comet assay.^19^ Protective role of TQ against As-induced toxicity in kidney have been demonstrated by Sener at el. They concluded that TQ can potentially be used as a remedial agent.^7^ Similarly, Fouad et al have reported that TQ not only decrease the As-induced expression of inducible nitric oxide (NO) synthase and caspase-3 but also considerably increased the level of serum testosterone and glutathione (GSH) and considerably diminished malondialdehyde (MDA) and NO levels caused from As administration in the testicular tissue.^6^ More recently TQ was reported to ameliorate the neurotoxic effect of As and through its antioxidant mechanism suppress the induced oxidative stress in the nervous system.^8^

### Protective roles against cadmium toxicity

Cd as naturally happening metal is extensively used in industries. Natural and anthropogenic activities can contaminate environment with Cd and stays intact for long periods of time (half-life is about 10-30 years). It can enter into the food chain via consumption of contaminated plants, fish, and animals. Food is a main pathway for human exposure to Cd (90%).^24^ Cd can be accumulated in some foods like shellfish/mollusks, animal offal, and certain mushroom and cereals in high concentration. Several side effects including kidney and bone damage upon chronic exposure to Cd was reported.^25^ The mechanisms of Cd toxicity include induction of oxidative, inflammatory response, endoplasmic reticulum stress, genotoxicity, and interfere with the functions of essential metal (specially zinc).^26^ According to the study of Zafeer et al, treatment with CdCl_2_ (5 mM) caused a considerable increase in protein carbonyl and reduction of GSH content.^9^ However, the histopathological studies of rat’s kidney showed that TQ noticeably decreased the Cd toxicity and protected against kidney histological damage. Also immunohistochemical analysis showed that TQ meaningfully decreased the Cd-induced over expression of nuclear factor-kB in renal tissue.^27^ Sayed et al have been shown that long-term treatment with low doses of Cd changed the antioxidant enzymatic profile and induced oxidative and spermatological damages.^28^ In addition, histopathological changes in testes, epididymis and accessory glands have been observed. However, the co-treatment with TQ exhibited favorable effect in all aforementioned parameters. It has been concluded that testes protection effect of TQ against the detrimental was due to its antioxidant and anti-inflammatory activities. Besides, free radical scavenging activity may be another protective effect of TQ.^29^

### Protective roles against Mercury toxicity

Hg and its organic and inorganic species are toxic compounds (the most toxic form is methylmercury [MeHg]) and human can exposure to mercury via consumption of food like fish.^30^ Half-life of Hg species are nearly 1–3 months, and can be eliminated mainly as MeHg in urine and as iHg (inorganic mercury) in faeces.^30^ Due to the volatile characteristics of Hg species, the inhalation of Hg vapor and its compounds have adverse effects on the nervous, gastrointestinal and immune systems, lungs and renal tissues. In addition, it can be changed into neurotoxic and teratogenic agents.^31^ TQ nephroprotective effect against inflammation and oxidative stress has been reported as well. For example, Fouda et al, revealed that the number of superoxide-positive cells decreased up to 70% upon treatment with TQ and proposed it as a protection method from mercury chloride–induced kidney failure.^10^ Another study confirmed that TQ improves the renal proliferative reaction and decreased histological injury like renal cell apoptosis and proliferative responses owing to Hg exposure in rats.^32^

### Protective roles against Lead toxicity

Pb is non-essential heavy metal with high toxicity potential. Pb in cereal and dairy products are of specific concern and are carefully checked by international organizations because these products are introduced in infants and children diets as the first solid food.^33^ It has several clinical adverse functions and the most important effect of it is on the oxidative stress mechanism, whereas antioxidants including GSH decrease ROS induced cell damage.^34^ According to current guidelines delivered by the Centers for Disease Control and Prevention (CDC), 10 μg/dL of blood Pb levels can be hazardous and proper treatment should be considered.^35^ TQ can be a promising substitute for the conventional therapeutic drugs due to its high pharmacological activity and low systemic toxicity.^36^ In this context, Mabrouk and Cheikh reported that supplementation with TQ significantly protected rat kidney against Pb-induced renal impairment.^11^ Another study showed that co-treatment of TQ with Pb acetate noticeably diminished the occurrence of Pb acetate-induced pathological injuries.^12^ In this context, Mahrous et al reported that TQ significantly reduced the harmful effect of Pb acetate in male rats through reducing DNA damage and alterations in the gene expression, levels of MDA and protein carbonyl (PC) and also increasing GSH levels.^37^

### Protective roles against aluminum toxicity

Al exists in air, water, and many foods but not in its metallic form. It is used in the food additive, medicines and cooking utensils.^38^ Neurotoxicity occurrence via treatment with Al was related to its high levels in brain and neurofibrillary tangles (NFT) that are known as symptom of Alzheimer’s disease.^39^ *N. sativa* and TQ have a protective effect against Al-induced cerebellar toxicity in animals. Kamal and Kamal reported that administration of Al in the rat cerebellum significantly reduced the number of Purkinje cells and induced some damages such as cytoplasmic vacuolation, dilatation of Golgi cisternae, and mitochondria with dilated cristae in Purkinje cells and administration of *N. sativa* with Al showed a noticeable protection against these alterations.^13^

### Protective roles against iron toxicity

Fe as the fourth most abundant element by weight constitutes the main part of earth crust. The Fe ions involve in biological systems through oxidation-reduction process of Fe^2+^ to Fe^3+^ or reverse and they are part of various chemical substances with a very important role in various organisms.^14^ Although the most common causes of anemia in human is deficiency in Fe level, high concentrations of Fe become toxic for cells and it can result in various side effects as well as arising risk of cancer, heart diseases, and other disorders. Protective effects of *N. sativa* in Fe intoxication experiment has been carried out. According to the study of Ahmadi et al, *N. sativa* can be effec­tive for health protection and improvement of rabbit’s organism, even with administration of high doses of Fe for a short period of time.^14^ Similarly, Kishwaret al demonstrated that TQ can successfully form complexes with trace metals such as Fe(III), Cr(VI), Cu(II), V(IV) and Co(II) in pH range of 3.00-4.00 as chelating agent in case of toxicity of aforementioned metals.^15^

### Protective roles against chromium toxicity

Cr is well known essential trace element for human metabolism that plays role in preserving of glucose, protein, and fat normal metabolism for the human. Cr as a seventh most abundant metal in the earth crust can pollute environment.^40^ The major Cr contamination can be through the leather tanning, electroplating, and stainless steel industries.^41^ DNA damage including the formation of DNA adducts and alteration in DNA replication and transcription owing to Cr administration has been confirmed by various studies. In addition, toxic, genotoxic, mutagenic and carcinogenic properties on human and animals has been reported.^42^ Several research studies have shown the TQ pharmacological activities and its other beneficial effects. Kishwar et al had reported that Cr(VI) toxicity can be eliminated through complex formation between the TQ and Cr(VI) or by reduction of Cr(VI) to Cr(III).^17^ Another study had demonstrated that treated with Cr (VI) caused a considerable raise in liver and brain mRNA level of cytochrome as CYP1A2, CYP3A, and CYP2E1 compared to the control group. On the other word, co-treatment with Cr(VI) and *N. sativa* oil or TQ showed that mRNA level of genes as compared to treatment with Cr(VI) alone reduced considerably and also has been shown a considerable amelioration in the histological images.^16^

### Protective effects of Nigella sativa and thymoquinone against antibiotics residue toxicity

The employ of veterinary drugs that prevent the growth of microorganisms as feed additives in food-producing animals has capacity to produce residues in animal derived products (meat different, milk and dairy products, eggs and honey) and can generate a potential health hazard for consumers including hypersensitivity reactions, antimicrobial drug resistance, toxicity, mutagenicity, teratogenicity, and carcinogenicity.^43^ In addition, various studies have shown that antibiotics can lead to mitochondrial dysfunction and oxidative stress in mammalian cells.^44^ It has been reported that TQ reduces oxidative damage induced by a different free radical producing agents including oxytetracyclin (OTC) and gentamicin (GEM). In this section, the effects of TQ against some antibiotics residue toxicity are discussed. Table 2 shows the effect of *N. sativa* and its constituent TQ against antibiotics residue toxicity.

**Table 2:** Protective effects of *Nigella sativa* and its constituent thymoquinone against some antibiotics residue toxicity in different tissues

**Food Toxins**	**Food Source**	**Targets**	**Treatment Conditions**	**Main Findings**	**Ref.**
OTC	Milk - meat	Hematotoxicity	*In vivo*, Pigeons black seed: level of 2.5% with OTC	Black seed completely blocked the elicited effects by OTC.	^45^
		hepato-renal toxicity	*In vivo,* rabbits *N. sativa* oil and ascorbic acid	Protective role of NSO against the toxic effects of OTC by their free radical scavenging and strong antioxidant activities	^46^
		Nephrotoxicity	*In vivo* rats *N. sativa* oil: 0.5, 1.0 or 2.0 mL/kg/d	*N. sativa* may be useful in ameliorating signs of GEM nephrotoxicity in rats	^47^
GEM	Milk - meat	Nephrotoxicity	*In vivo* rats *N. sativa*: 0.2 -0.4 mL/kg	*N. sativa* prevents the toxic effects of GEM on the biochemical and histopathological parameters.	^48^
			*In vivo*, rabbit, Nigella oil: 2 mL and vitamin C: 250 mg	Decreasing oxidative stress and ability to prevent the energy decline in kidney tissues.	^49^

Abbreviations: OTC, oxytetracyclin; GEM, gentamicin; NSO, *Nigella sativa* oil.

### Protective effects on oxytetracyclin toxicity

OTC as one of the tetracyclines antibiotics is used extensively not only in human but also in veterinaries and meat industry. It also is used as feed additive or in drinking water to preserve ideal animal health for food production.^50^ For veterinary animals like poultries, cows, pigs and sheep, it is used to treat pneumonia, enteritis, septicemia, endometritis, metritis mastitis and other secondary bacterial infections.^50^ Excessive use of antibiotics in the treatment of dairy cows may result in antibiotic remaining in the milk and consumption of milk with high levels of OTC by humans leads to side effects including allergic responses, increase of bacterial resistance, the development of teratogenicity risk in the first trimester of pregnancy, the color change of the primary and permanent teeth.^50^ A recent study has reported that administration of OTC to pigeons meaningfully reduced total leukocyte and lymphocyte counts, raised lymphocyte, heterophil ratio, lysosomal enzyme activity and diminished reticuloendothelial system utility compared to controls. Co-administration of black seed with OTC perfectly inhabited the effects provoked by OTC and made immunostimulant effects in pigeons.^45^ In a similar study, Abdel-Daim reported that OTC leads in considerable modifications in serum biochemical renal-hepato hurt markers, and significantly inhibited the tissue antioxidant biomarkers and renal-hepatolipid peroxidation in animal treated with it. However, combination of *N. sativa* oil (NSO) with OTC protects animal against OTC induced serum and tissue biochemical revisions. Moreover, NSO has been hepatoprotective and antioxidant attributes and it has been shown that the toxic effects of OTC can be protected by NSO via their free radical scavenging and strong antioxidant activities.^46^

### Protective effects on Gentamicin toxicity

GEM is an effective antibiotic against gram-negative bacteria that cause infection in human and animals. It is used for the treatment of mastitis in the dairy cow and often appears in raw milk.^51^ Nephrotoxicity and renal failure are main side effects of its remedial doses. Some studies showed that GEM caused proximal tubular injury, glomerular, tubular necrosis, interstitial nephritis and desquamation of the tubular epithelial cells in rat’s kidney. *N. sativa* increased GSH and total antioxidant status (TAS) in renal cortex and improved the histological and biochemical effect of GEM. A similar study has shown administration of *N. sativa* and GEM compared to the group treated with just GEM caused significant reductions in MDA level and nitric oxide production and rises superoxide dismutase (SOD) and glutathione peroxidase (GPx) activities. In addition, *N. sativa* improves induced oxidative stress.^48^ Moreover, Saleem et al have been evaluated the synergistic nephroprotective effects of NSO and vitamin C and reported that they reduced levels of nephrotoxicity indicators like; serum creatinine, blood urea nitrogen (BUN), and antioxidant activity compared to GEM treated rabbits.^49^ A recent study has shown that GEM could significantly raise the levels of creatinine, BUN, thiobarbituric acid-reactive substances (TBARS) and total nitrate/nitrite (NOx) and contrarily significantly reduced the levels of GSH, catalase (CAT), ATP in renal tissues. Moreover, TQ inhibited not only the increase of TBARS, BUN, creatinine and NOx but also the decrease of CAT, GPx, GSH, and ATP in GEM-treated rats compared to untreated group. In addition, histopathological inspection of renal tissues showed that TQ supplementation can inhibit the increase of GEM-induced nephrotoxicity in rats.

### Protective roles of Nigella sativa and its constituent against pesticides toxicity

Pesticides are produced to control or remove pests (weeds, insects, or other organisms that interfere with human activity) but they can also be toxic (poisonous) to plants and animals as well as humans. Pesticides toxicity is dose dependent and can be either acute or chronic including oral, dermal or inhalation poisoning, carcinogenic, teratogenic, mutagenic and reproductive toxicity.^43^ Numerous plant natural products are accessible for reduction of toxicity effects of pesticides in the environment and human. NSO or its active ingredient TQ, can decrease toxicity of pesticides. For example, Mostafalou et al. have confirmed protective role of TQ against Malathion induced disruption in isolated pancreatic islets of dog.^52^ In this section, several studies, which investigated protective effects of TQ against pesticides induced toxicities are introduced as follows. In addition, Table 3 shows the effect of *N. sativa* and TQ against pesticides toxicity.

**Table 3:** Protective effects of *Nigella sativa* and its constituents against various pesticides toxicity in different tissues

**Food toxins**	**Food Source**	**Targets**	**Treatment Conditions**	**Main Findings**	**Ref.**
ACMP	Leafy vegetables, citrus fruits, pome fruits, grapes, cotton, Cole crops, cucumber, potato, tomato, eggplant cotton, corn, almonds and fruit trees including oranges, bananas, and apples	Reproductive toxicity	*In vivo*, rat, NSO: 1 mL/kg/bw)	NSO protects against reproductive toxicity of ACMP.	^53,54^
CPS		Reproductive toxicity, hormonal alterations, and oxidative damage	*In vivo*, rat, NSO: (1 mL/kg/d)	NSO can improve semen picture and moderate CPS-induced reproductive toxicity.	^55^
IC		Immunological and histological changes	*In vivo*, rat, TQ: 1 mg/kg/d	TQ has been able to improve toxicity due to decreasing oxidative.	^56^
		Oxidative stress (blood, liver, kidney, and heart)	*In vivo*, mice, TQ: 10 mg/kg/d	TQ protect mice against IC-induced oxidative stress.	^57^
DI		Hematotoxicity, genotoxicity, immunotoxicity	*In vivo*, rat, TQ: 2.5, 5, 10 mg/kg/d	Metabolism of the TQ antioxidant properties caused reduction in toxicity.	^58^
		Brain damage	*In vivo*, rat, TQ: 10 mg/kg/d	The intoxication decrease was due to antioxidant potential of TQ	^59^
PPr		Brain regions	*In vivo*, rat NSO: 1 mL/kg/bw/d	NSO significantly reduces PPr-induced oxidative stress in rat brain regions via a free radicals scavenging mechanism	^60^

Abbreviations: ACMP, acetamiprid; NSO, *Nigella sativa* oil; CPS, chlorpyrifos; TQ, thymoquinone; IC, imidacloprid; DI, diazinon; PPr, propoxur.

### Protective roles against acetamiprid toxicity

Acetamiprid (ACMP) is an odorless organic neonicotinoid insecticide and has systemic effect on control of sucking insects on crops including leafy vegetables, citrus fruits, pome fruits, grapes, cotton, Cole crops, cucumber, potato, tomato, eggplant, Japanese radish and ornamental plants.^61^ ACMP did not show any carcinogen effects on human while it has a low chronic and acute toxicity in animals with no sign of carcinogenicity, mutagenicity or neurotoxicity.^62^ A recent study has indicated ACMP as a cause of sexual dysfunction in males that may be associated with the problem of decreasing fertility.^63^ ACMP can lead to reproductive toxicity in male rats by a decrease in body weight gain, relative weights of reproductive organs (testis, epididymis and seminal vesicle), spermatids number, sperm count, sperm motility, and testosterone levels. It also has induced histopathological changes including tubular atrophy, disorganization, and degenerative aspect of the seminal epithelium in some seminiferous tubules marked by spermatogenesis perturbation and poor sperm and presence of sloughing cell debris in their lumens. It is revealed that NSO co-administration modulated ACMP-induced reproductive side effects. This protective role may be due to antioxidant effects and the ability to reduce TBARS levels.^53^ Additionally, a similar study has reported that ACMP meaningfully reduced the body weight gain and the total weights of reproductive organs (epididymis, testes, and seminal vesicles). Besides, the failure in spermatids number, sperm count, sperm motility and testosterone level as well as the increased TBARS level and dead sperm were the least significant modifications in semen characteristics of ACMP group. Treatment with NSO alone may significantly incite the enhancement of spermatids number, spermatogenesis, and the weight of seminal vesicles. On the other hand, the co-administration of NSO along with ACMP can more efficiently modulate the ACMP-induced harmful effects on reproductive organs weights, semen quality, testosterone, and TBARS levels. Obviously, the protective role of NSO against ACMP- induced reproductive toxicity may be due to its antioxidant properties and capability to decrease TBARS level.^54^

### Protective roles against chlorpyrifos toxicity

Chlorpyrifos (CPS) is an organophosphate pesticide that applied for destroying of pests like worms and control of insects in agricultural, residential and commercial settings.^64^ WHO introduced CPS as a relatively hazardous substance to humans and reported that exposure to CPS during pregnancy can damage the mental development of fetus.^65^ Recently, the studies have reported that CPS can induce side effects on male rat reproductive system by a decrease in sperm count and production, body weight, sexual hormones, and relative weight of reproductive organs accompanied by the increase in dead and abnormal sperms. All altered semen parameters normalized in rats treated with CPS during treatment with NSO and its protective effect can be because of its antioxidant capacity.^55^

### Protective roles against imidacloprid toxicity

IC as a member of the neonicotinoid insecticide class is highly effective on different insects.^66^ Prevention of termite damage, pest control for gardens and turf, fleas control in treated domestic pets and protection of trees from boring insects are among other examples of this pesticides application. It can be considered as an “unlikely” carcinogen and a weakly mutagenic by the United States. Environmental Protection Agency (EPA) (group E).^67^ Recent studies have shown that IC significantly increases the total leukocyte counts, (especially IgGs), the hemagglutination of antibodies, MDA, alanine transaminase (ALT), aspartate aminotransferase (AST), alkaline phosphatase (ALP), and total immunoglobulins (Igs) in comparison with the untreated control group. TQ could decrease the toxicity of IC by declining oxidative stress and improving immune efficiency.^56^ Similarly, Ince et al demonstrated that administration of TQ reduced IC-induced oxidative stress, LPO, and activities of the antioxidant enzymes. Besides, TQ had protective effect on the IC-induced oxidative stress and histopathological alterations in tissues of mice by improving antioxidant protection mechanisms.^57^

### Protective roles against diazinon toxicity

Diazinon (DI) as a non-systemic organophosphate insecticide can be utilized for the control of agricultural, and domestic pests. The acute and chronic toxicity of DI has been approved in humans and animals.^68^ Recent studies indicated that acute and chronic toxicity of DI induces oxidative stress and leads to the production of free radicals. The production of ROS induces oxidization in various biomolecules such as membrane lipids, proteins and nucleic acids and alters the activity of antioxidants enzymes or ROS scavenging enzymes in animals.^69^ Danaei et al have shown that DI reduced red blood cells (RBCs), white blood cells (WBCs), hemoglobin, hematocrit, platelets, butyrl- and acetylcholinesterase function and interferon gamma and enhanced the micronucleus index of interleukin 10 and interleukin 4 in comparison with the control group. Co-treatment with TQ and DI decreased hematotoxicity and immunotoxicity but did not inhibit genotoxicity very well. Also, it has showed that TQ had not considerable effect on genotoxicity but it reduced the hematological toxicity, immunotoxicity, butyrl- and acetylcholinesterase activity in DI treated rats.^58^ Additionally, TQ reduces nitrous oxide and significantly increases SOD in damaged brain of rats that induced with DI.^59^ The successful protective effect of TQ on DI toxicity can be related to the antioxidant properties of its main component.

### Protective roles against propoxur toxicity

Propoxur (PPr) is a carbamate insecticide with long residual effect used against forestry, household pests, turf, and fleas. PPr exerts neurotoxic effects through the massive reversible inhibition of acetylcholine esterase (AChE). Consequently, the health risk of human is high due to the exposure by frequent low doses of these materials. The exact mechanism of PPr-induced toxicity is not being completely defined. Involvement of ROS may be a probable mechanism of PPr-induced toxicity. PPr treatment considerably raised the levels of protein carbonyl content (PCC), LPO and oxidized glutathione (GSSG) in brain areas while levels of GSH and the activities of CAT, SOD, GSH-Px, GST (glutathione-S-transferase) and AChE were remarkably reduced. Administration of PPr with NSO repaired such biochemical factors to the control levels except for GST activity. NSO meaningfully decreased oxidative stress induced in rat brain areas through a free radicals scavenging mechanism that emphasized its antioxidant activity.^60^

### Protective roles of Nigella sativa and thymoquinone against food process toxicants

Food processing and preservation have played important roles in achieving food sufficiency for the human being.^70^ Food processing and cooking can generate toxic compounds in food as a naturally-occurring constituent or form as the result of handling or processing such as N-Nitrosamines, Acrylamide, polycyclic aromatic hydrocarbons (PAHs), phenolic compounds, heterocyclic aromatic amines (PhIP), lipid polymerisation products resulting from deep-fat frying, lipid oxidation products, Maillard-browning products, ethylcarbamate and furan.^70,71^ Also, Bisphenol A (BPA) can produce during food packaging.^72^ A number of studies have reported that all food process toxicants can induce toxicity in animals and humans.^73^ TQ has displayed different properties in modern pharmacology and scientific findings and can reduce toxicity effect of food process toxicants.^73^ The present part was undertaken to overview the protective effect of TQ on some well-known food process toxicants. Table 4 shows the effect of *N. sativa* and TQ against food processing toxicants.

**Table 4:** Protective effects of *Nigella sativa* and its constituents against some food process toxicants in different tissues

**Food toxins**	**Food source**	**Targets**	**Treatment Conditions**	**Main Findings**	**Ref.**
DENA	Fried foods, cosmetic products, tobacco smoke, cheddar cheese, and pesticides.	Erythrocyte fragility	*In vivo*, rat TQ:4 mg/kg/5 days/p.o.	-	^74^
		Hepatic carcinogenesis	*In vivo*, rat TQ: (4 mg/kg/d)	TQ decreases oxidative stress and preserves both the activity and mRNA expression of antioxidant enzymes	^75^
ACR	Making paper, dyes, plastics, and in treating drinking water and wastewater. caulk, food packaging,	Neurotoxicity	*In vivo*, rat TQ: 2.5, 5, 10 mg/kg IP	Neuroprotective effect of TQ in this model due to the antioxidant activity	^76^
			Vitamin E: 200 mg/kg/d orally TQ:5 mg/kg/d, ip	The neurotoxicity decrease was due to antioxidant potential of TQ	^77^
B[a]P	Coal tar, tobacco smoke, and grilled meats	Forestomach carcinogenesis	*In vivo*, mice 0.01% of TQ in drinking water	Detoxification processes of TQ may be through its antioxidant and anti-inflammatory activities.	^78^
		Mutagenic effect in murine bone marrow cells	*In vivo*, mice 0.01% of TQ in drinking water	TQ can inhibit the cytotoxic effects of exposure to the carcinogen B[a]P in initial phases.	^73^
		Genotoxicity	*In vitro*, cultured human lymphocytes TQ: TQ ranging from 0.625, 1.25, 2.5, 5, 10 µM	TQ can reduce DNA damage.	^79^
BPA	Baby bottles, and beverage containers	Reproductive system	*In vivo*, mice TQ: 10 mg/kg/d	TQ ameliorates these toxic effects	^80^
		Hepatotoxicity	*In vivo*, rat, TQ: 10 mg/kg/d	TQ reduces elevated levels of hepatic biomarkers and decreases lipid peroxidation	^81^

Abbreviations: DENA, diethylnitrosamine; TQ, thymoquinone; ACR, acrylamide; B[a]P, banzo[a]pyren; BPA, Bisphenol A.

### Protective roles against diethylnitrosamine toxicity

Diethylnitrosamine (DENA) as a chemical toxin can be generated in fried foods, tobacco smoke, cheddar cheese, pesticides and cosmetic products. DENA is well-known as a carcinogenic and in addition to hepatocarcinogenic effect, it can lead to different gastrointestinal cancers like gastric cancers, esophagus.^82^ A study has shown that DENA significantly increased ALP, total bilirubin, TBARS, ALT, total NOx, GSH, GST, GPx and CAT activity in hepatic tissues and led to severe histopathological alterations in hepatic tissue. Moreover, TQ supplementation could be ameliorated biochemical and histopathological alterations induced by DENA to the same value of control case. DENA induced initiation of liver carcinogenesis through reduced mRNA expression of GPx, CAT and GST. TQ inhibited the development of DENA-mediated primary hepatic cancer by declining oxidative stress and protected both the function and expression of antioxidant enzymes at mRNA level.^75^ Also in a recent study by Amin et al, enhancements in erythrocyte, hematocrit, and hemoglobin count have been seen in DENA-induced rats in which the effect of TQ on these markers was not statistically significant.^74^

### Protective roles against acrylamide toxicity

Acrylamide (ACR) has wide usage in different industries such as fabricating paper, dyes, and plastics, and in treating drinking water and wastewater. There are low amounts of ACR in several products like caulk, food packaging and some adhesives. ACR is found in cigarette smoke as well.^83^ ACR can also form in some starchy foods during high-temperature cooking as well as roasting, frying, and baking. The U.S. government agencies considered ACR as a potential occupational carcinogen and it is categorized as a Group 2A carcinogen by the International Agency for Research on Cancer (IARC).^84^ It also forms in food during process heating. A critical role of oxidative stress in ACR-induced neurotoxicity in both *in vitro* and *in vivo* models has been demonstrated. Protective effects of TQ against ACR induced neurotoxicity in experimental animal models have been reported. Mehri et al reported that TQ supplementation significantly decreased ACR induced gait abnormalities in Wistar rats.^76^ Also in a similar study by El-Aaron combined treatment of ACR with TQ or vitamin E reduced the neurotoxicity.^77^

### Protective roles against benzo[a]pyrene toxicity

Banzo[a]pyren (B[a]P)^46^ is a PAH. Coal tar, tobacco smoke and many foods, particularly grilled meats may contain ubiquitous compound. Its diol epoxide metabolites (more commonly known as BPDE) result in mutations and eventually cancer due to interaction with DNA. It is classified as a Group 1 carcinogen by the IARC.^85^ Treatment with TQ alone indicated a significant inhibition of the enzyme activities of liver GST and DT-diaphorase. Moreover, administration of TQ in combination with B[a]P resulted in normal enzyme actions and significant reduction in liver lipid peroxides.^78^ Previous studies investigated that daily intake of TQ significantly decreased the frequencies of chromosomal aberrations and damaged cells compared to the clastogenic activity of B[a]P alone in mouse bone marrow cells.^73^ The results of studies suggested the importance of TQ as a natural dietetic supplement for counteracting the cytotoxic effects of the mutagen B[a]P in primary phases. Co-administrated with TQ and B[a]P moderated LPO and GSH levels in normal rat liver.^78^ The possible chemoprotective effect of TQ against B[a]P-induced chromosomal aberrations (CAs) in mouse bone marrow cells was described. Daily intake of TQ significantly decreased the frequencies of CAs and injured cells related to the clastogenic activity of group treated with B[a]P alone.^73^ Hussein reported that TQ treatment was able to mitigate the induced lung cancer by B[a]P through enhancing the levels of serum carcinoembryonic antigen (CEA), haptoglobin (HPT), adenosine deaminase (ADA) and gamma glutamyltransferase (ɤ GT) as well as increasing the expression of caspase 3 gene, DNA fragmentation, cycloxygenase-2 (COX-2) and L-malondialdehyde (L-MDA) in lung tissues.^86^ It could be concluded that TQ may be effective in decreasing lung cancer by its radical scavenging activity, anti-inflammatory effect, regenerating endogenous antioxidant mechanisms, decreasing caspase-3 gene, and DNA damage in lung tissues.

### Protective roles against bisphenol A toxicity

Bisphenol A (BPA) is a broadly used compound in resin and plastic industry and is the main constituent of plastic baby bottles, food containers and children’s toys.^87^ Exposure of BPA led to various health problems including cardiovascular disease, neurobehavioral disorders, metabolic disorders, abnormalities of the reproductive system, and cancer.^88^ A recent study has shown that administration of BPA significantly decreased seminiferous tubules diameter and epithelial height with impaired spermatogenesis, plasma testosterone, levels of luteinizing hormone (LH) and follicle-stimulating hormone (FSH), androgen receptors. Administration of TQ with BPA led to improvement of these side effects.^80^ BPA has a wide range of side effects and can cause oxidative stress in hepatic cells.^89^ Decrease in hepatocytes viability and their mitochondrial action have shown in the rats treated with BPA.^90^ Administration of TQ greatly normalized suppressed enzymatic and non-enzymatic antioxidants such as GSH, GPx, GST, SOD, and CAT. It also reduced elevated levels of hepatic biomarkers and decreased LPO.^81^

### Protective roles of Nigella sativa and thymoquinone against mycotoxins toxicity

Mycotoxins like aflatoxins, ochratoxins, patulin, trichothecenes, zearalenone, fumonisins, tremorgenic toxins, and ergot alkaloids are secondary metabolites of fungus which not only have harmful effects on living creatures (humans, animals), and economic losses (in crops) but also cause in humans and animals death and illness.^91,92^ Consumption of food that contaminated via mycotoxin cause both short-term and long-term toxicity in human and animals. Mycotoxins, especially aflatoxins (AFB1) have acute toxicity and chronic carcinogenicity, teratogenicity, hepatotoxicity, mutagenicity, nephrotoxicity and immunosuppressive effects. The human disease acquired by eating the different agricultural commodities infected by mycotoxins including oilseeds, tree nuts, cereals, spices and dairy products.^91,92^ *N. sativa* has been considered as a natural medicine to treat various poisoning or disorder in liver and kidney function induced by mycotoxins. The anti-yeast activity of TQ was evaluated by various researchers against some dairy spoilage yeast species.^93^ This section was undertaken to evaluate the TQ protective effects against mycotoxins-induced acute and chronic toxicity. Table 5 shows the efficacy of *N. sativa* and TQ against mycotoxins toxicity.

**Table 5:** *Nigella sativa* and its constituent thymoquinone against some mycotoxins toxicity in different tissues

**Food Toxins**	**Food Source**	**Targets**	**Treatment Conditions**	**Main Findings**	**Ref.**
ZEN	Corn, wheat, barley, oats, sorghum, and sesame, cereals, peanuts, dried vine fruits, cocoa beans, feedstuff, green coffee beans, wine grapes, poultry feeds and beer.	Nephrotoxicity	*In vivo*, mice TQ: (10 mg/kg b.w)	TQ can enhance kidney cells detoxification of ZEN mycotoxin.	^94,95^
Ochratoxin A		Toxicity in Liver and kidney	*In vivo*, rat NSO: (0.3 mL/d)	NSO can reduce the toxic effect of OTA on liver and kidney tissue.	^96^
VJ		Hepatotoxicity	*In vivo*, rat NSO:800 mg/kg b.w	NSO leads to adsorption of the toxic substance.	^97^
		toxicity	*In vivo*, mice TQ: 4.5, 9 and 18 mg/kg	TQ can increase resistance to oxidative stress and reduce LPO.	^98^
AFB1		Hematological and biochemical changes	*In vivo*, rat NSO: 5 mg /kg/body wt. *Syzygium aromaticum* oil: 5 mg /kg/body	NSO was found to be more effective in restoring the parameters that were altered by AFB1 in rats.	^99^

Abbreviatopns: ZEN, zearalenone; LPO, lipid peroxidation; OTA, ochratoxin A; VJ, verrucarin J; AFB1, aflatoxin B1; NSO, *Nigella sativa* oil.

### Protective roles against zearalenone toxicity

Zearalenone (ZEN) is an example of mycotoxin made by *Fusarium* species with estrogenic and anabolic activity. It is one of the most extensively dispersed *Fusarium* mycotoxins that is observed at high occurrence in many important corps likes corn, wheat, barley, oats, sorghum and sesame and infected human and animal upon their consumptions.^100^ Nephrotoxicity of ZEN was evaluated in mice by given single and repeated doses of ZEN mycotoxin via the oral route. To overcome renal toxicity of ZEN, BUN and alpha-fetoprotein (AFP) concentration change was evaluated. The attained results showed that BUN and AFP concentration were remarkably decreased when mice treated with combination of TQ and ZEN in comparison with mice treated with ZEN alone. In addition, a highly significant increase in the blood creatinine and pyruvate kinase isoenzyme tumor M2 concentration was observed in TQ and ZEN treated group (T4) compared to control group. There was no significant difference in TAS concentration between T4 and control group while a highly significant reduction was detected between T4 and T3 groups. It should be mentioned that histological changes of mice kidney coincided with biochemical changes.^94^

### Protective roles against ochratoxin A toxicity

Ochratoxin A (OTA) is a naturally occurring mycotoxin generated by *Aspergillus*,^101^ which has been found in a variety of foodstuffs such as cereals, peanuts, dried vine fruits, cocoa beans, feedstuff, green coffee beans, wine grapes, poultry feeds and beer.^102^ OTA is classified by IARC of WHO as a probably carcinogenic agent to humans.^103^ It is carcinogenic, teratogenic, nephrotoxic, hepatotoxic agent to animals and possibly to humans.^104^ Alhussaini and AL-Yahya have examined the histological alterations in the kidney and liver tissues of rats treated with OTA and OTA + NSO. Significant histological alterations have revealed in the liver and kidney tissues of rats treated with OTA. In liver tissue, it led to vacuolar deterioration and necrosis of the liver cells, central vein dilation and sinusoidal, bile duct replication, congestion of portal vein and dilation. Also cellular infiltration, slight rise in the collagen fibers, reduction in carbohydrates and protein were detected. In the kidney tissue, OTA resulted in necrosis in the nuclei, tubular epithelial cells deterioration, decline in the carbohydrates and protein and fibrous tissue replication. However, NSO significantly decreased the severity of injuries induced by OTA. Also administration of NSO with OTA considerably decreased the toxic effect of OTA on liver and kidney tissue of rats.^96^

### Protective roles against verrucarin J toxicity

Verrucarin J (VJ) belongs to trichothecene family of mycotoxins that generated in different cereals such as wheat, oats or maize by several species of *Trichothecium*, *Fusarium, Trichoderma, Myrothecium, Cephalosporium*.^105^ Some of molds that generate trichothecene mycotoxins like *Stachybotrys chartarum* can growth in wet environments and causes health problems among residents of the building.^106^ VJ also can infect foods of animals and humans. A recent study has reported that VJ can raise levels of SOD, TBARs and 5-nucleotidase in the renal and blood tissue of the rats. Treatment of hepatic tissue with VJ reduced levels of zinc and glucose in blood, which in turn decreased the GSH and glucose-6-phosphate dehydrogenase level and this problem can be ameliorated by NSO. It has been found that treatment of rats with NSO may improve the harmful effects of toxin and the results suggested the utility of NSO as an antidote and antioxidant protectant against VJ in rats.^107^

### Protective roles against aflatoxin B1 toxicity

Aflatoxin B1 (AFB1) is an aflatoxin created by *Aspergillus parasiticus* and *Aspergillus flavus*. AFB1 is commonly found in a variety of foods such as peanut, a promise of cotton, corn and other seeds and animals feed.^108^ It is an extremely potent carcinogen with a TD50 of 0.0032 mg/kg/d in animal models and it seems that rats are much more sensitive than other animals like monkey.^109^ AFB1 is the most toxic aflatoxin in hepatocellular carcinoma (HCC). Similarly in animals, AFB1 has been revealed to be mutagenic, teratogeni and exhibit immunosuppressive properties.^110^ TQ showed a protective effect against AFB1-induced hepatotoxicity in mice by decrease of liver hurt indicators including AST, ALT, and ALP and also via inhibiting degradation and necrosis of liver tissue. MDA which is a marker of LPO was increased in AFB1-intoxicated mice in liver, while pretreatment with TQ significantly prevented MDA production. Histopathological effects such as inflammation, necrosis, disruption of hepatocytes, hyperplasia of Kupffer cells, and infiltration of mononuclear cells and increased in size of hepatocytes were measured as signs of toxicity. TQ reduced the number of inflammatory cells and ameliorated the histopathological changes.^98^ In a similar study, it has been shown that treatment with NSO and *Syzygium aromaticum* oil have protective efficacy against aflatoxin contamination in rats and NSO was found more efficient in rats protection and it significantly improved the biochemical parameters affected by aflatoxin.^99^

### Protective roles of Nigella sativa and TQ against food additives toxicity

Food additives are natural or synthetic constituents added to foods to carry out specific technological functions. There are around 3000 food additives that allowed for use in food industry and are divided by 6 main parts including preservatives, nutritional additives, coloring, flavoring and texturing agents. Also, other various agents which can be mentioned are enzymes, chelating agents, antifoaming agents, surface finishing agents.^111-114^ Some studies have shown the sub-chronic, chronic and acute toxicity of various food additives in human and animals. TQ possess beneficial effects against toxicity that can occur via food additives.^115^ In this part the protective effects of N. sativa and its main component against toxicity of food additives has been discussed. Table 6 shows the effect of *N. sativa* and its active ingredient TQ against food additives toxicity.

**Table 6:** Protective effects of *Nigella sativa* and its constituents against toxicity of 2 food additives in different tissues

**Food Toxins**	**Food Source**	**Targets**	**Treatment Conditions**	**Main Findings**	**Ref.**
TBHQ	Vegetable oils, numerous edible animal fats, and meat products	Hepatotoxicity	*In vivo*, rat TQ: 1 mM	TQ protects the liver enzymes leakage.	^116^
Carrageenan	Thickening, gelling and stabilizing abilities	Inflammatory	*In vivo*, rat TQ: 500 mg/kg body weight	TQ inhibits carrageenan-induced paw edema in a dose-dependent manner.	^117^
		Inflammatory	*In vivo*, rat extract of *N. sativa*: 250, 500 mg/kg body weight	*N. sativa* inhibits inflammation and pain.	^118^

Abbreviations: TBHQ, Tert-butylhydroquinone.

### Protective roles against Tert-butylhydroquinone toxicity

TBHQ is an effective preservative, which has been used as an antioxidant for unsaturated vegetable oils, many eatable animal fats and meat products at concentrations less than 0.02%.^119^ TBHQ in high doses has some adverse effects on animal model including DNA damage and stomach tumors and in addition to leakage of cytosolic enzymes. It can also be hepatotoxic agent which in case of exposure stimulates rapid oxidation of intracellular GSH and pyridine nucleotides.^120^ TQ not only could increase viability of hepatocytes in TBHQ treated rat but also decrease the leakage of ALT and AST.^116^

### Protective roles against Carrageenan toxicity

Carrageenan as a sulphated linear polysaccharide of D-galactose and 3,6-anhydro-D-galactose can be attained from certain red seaweeds of the Rhodophyceae class. Carrageenans are generally used in food industry owing to their exceptional physical functional properties like thickening, gelling, emulsifying and stabilizing abilities. They also have been utilized for improvement of the texture of cottage cheese, control of the final texture in most dairy desserts and pudding, and as stabilizers for production of sausages and also in the meat-processing industry. Extract of black seed was studied for its anti-inflammatory activity in carrageenan-induced rat paw edema model. Dose-dependent and remarkable anti-inflammatory properties were reported. Therefore, extract of black seed could inhibit carrageenan-induced inflammation in rats.^118^ A similar study reported that *N. sativa* reserved carrageenan-induced paw edema in a dose-dependent manner as well.^117^

## Conclusion

Recently, some of the natural herbs and their bioactive components have been used in several studies with the purpose of toxicity prevention in different tissues induced by different chemical and natural toxins especially toxins in food due to daily intake. The accessibility and cost benefit properties and less toxic effects of natural plant constituents compared with synthetic products make them an ideal candidate for inhibition of food and chemical toxicity. This review summarized several *in vivo* and *in vitro* studies in order to realize the role of *N. sativa* and its bioactive component, TQ, in inhibition of food toxins related toxicities in different tissues. Heavy metals, antibiotics residue, food processing toxicants, mycotoxins and food additives are examples of natural and chemical toxic agents in foods that can be prevented by *N. sativa* and TQ. *N. sativa* and its bioactive components could protect different tissues against food toxins through numerous mechanisms as well as free radical scavenging, anti-inflammatory, antioxidant, amelioration in the disturbed levels of biochemical indicators, modulation of antioxidant defense systems, prevention of apoptosis and controlling effects on genes expression, and different signaling pathways. *N. sativa* has also ability to protect different organs and tissues such as kidney, liver, gastrointestinal, lung, heart, blood, brain, and reproductive system against toxins. In conclusion, based on the present review, *N. sativa* has a wide spectrum of protective activities against food toxicants induced toxicities. Therefore,* N. sativa* can be introduced as a supplement in the daily diet of individuals for inhibition of food toxins side effects. Also since there are not enough clinical trial studies on human, further investigations are required to determine the efficacy of natural products as a protective agent in human intoxication.

## Ethical Issues

Not applicable

## Conflict of Interest

The authors declare that they have no conflict of interests.

## Acknowledgments

The authors gratefully acknowledge the financial support of this study by the Tabriz University of Medical Sciences, Tabriz, Iran.
